# Supplementary material for: The interactome of intact mitochondria by cross-linking mass spectrometry provides evidence for coexisting respiratory supercomplexes
Source: Mol Cell Proteomics. 2017 Dec 8;17(2):216–32. doi: 10.1074/mcp.RA117.000470 (PMC5795388; doi:10.1074/mcp.RA117.000470)
Supplement: Supplemental Data [file supp_RA117.000470_133922_0_supp_23978_fzffwf.zip › spectra_annotation/mito_spectra_annotation/337-1-6-1-5-1.pdf]

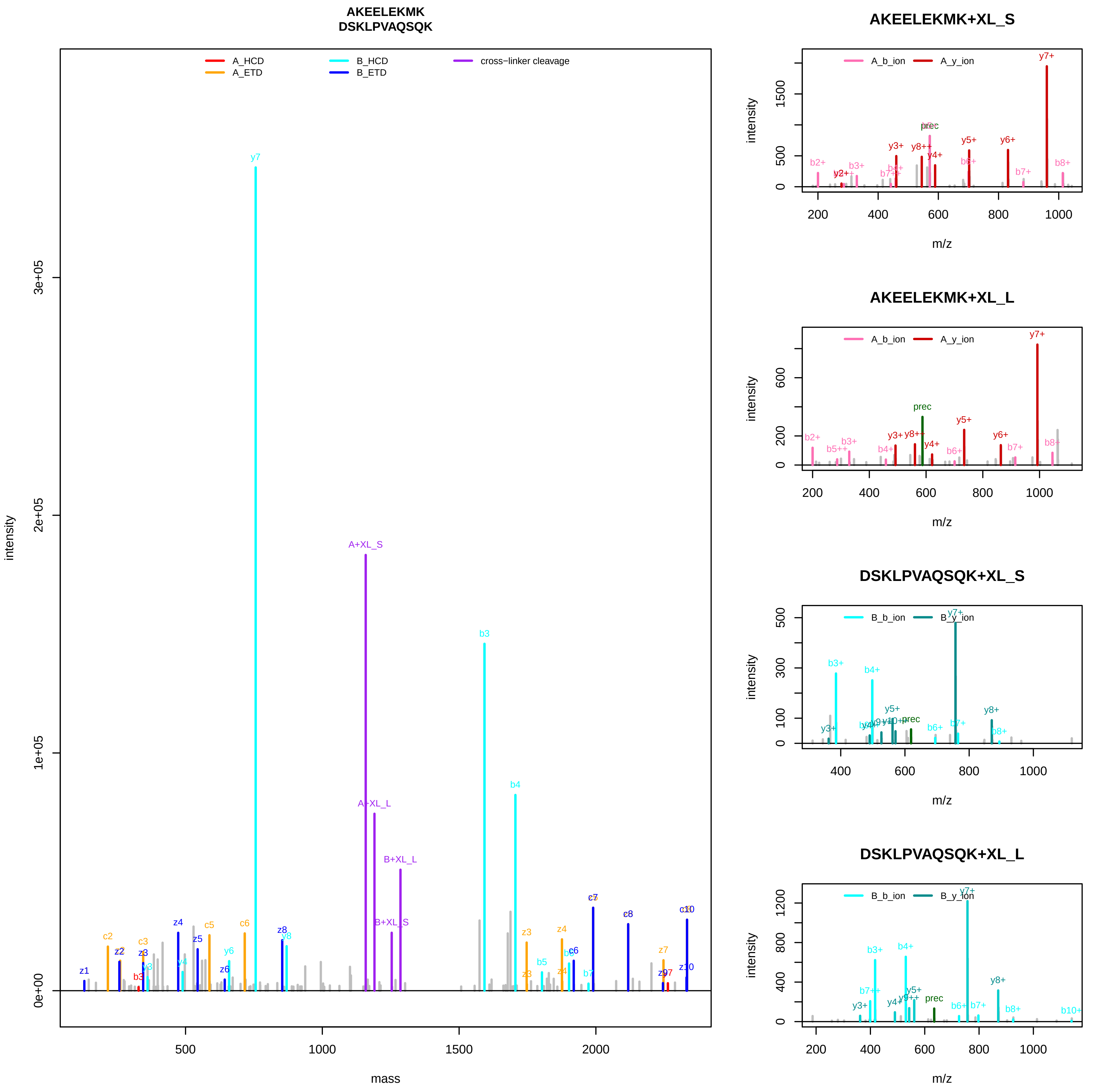

AKEELEKMK
DSKLPVAQSQK
A_HCD
A_ETD
B_HCD
B_ETD
cross−linker cleavage
y7
A+XL_S
A+XL_L
B+XL_L
B+XL_S
b3
b4
c2
c3
c5
c6
z1
z2
z3
z4
z5
z6
z8
y4
y6
y8
b5
z3
z4
c6
c7
c8
c10
z7
z10
intensity
0e+00
1e+05
2e+05
3e+05
500
1000
1500
2000
mass
AKEELEKMK+XL_S
A_b_ion
A_y_ion
y7+
prec
y3+
y8++
y4+
y5+
y6+
b2+
b3+
b7+
b8+
intensity
m/z
0
500
1500
200
400
600
800
1000
AKEELEKMK+XL_L
A_b_ion
A_y_ion
y7+
prec
y5+
y6+
y3+
y8++
y4+
b2+
b3+
b5++
b4+
b6+
b7+
b8+
intensity
m/z
0
200
600
200
400
600
800
1000
DSKLPVAQSQK+XL_S
B_b_ion
B_y_ion
y7+
b3+
b4+
y5+
prec
y3+
y8+
b6+
b7+
b8+
intensity
m/z
0
100
300
500
400
600
800
1000
DSKLPVAQSQK+XL_L
B_b_ion
B_y_ion
y7+
b3+
b4+
b7++
y5+
y3+
y4+
y9++
prec
b6+
b7+
y8+
b8+
b10+
intensity
m/z
0
400
800
1200
200
400
600
800
1000
